# The Force Generation in a Two-Joint Arm Model: Analysis of the Joint Torques in the Working Space

**DOI:** 10.3389/fnbot.2018.00077

**Published:** 2018-11-23

**Authors:** Alexander I. Kostyukov, Tomasz Tomiak

**Affiliations:** ^1^Department of Movement Physiology, National Academy of Sciences, Bogomoletz Institute of Physiology, Kyiv, Ukraine; ^2^Unit of the Theory of Sport and Motorics, Chair of Individual Sports, University of Physical Education and Sport, Gdańsk, Poland

**Keywords:** motor control, electromyography, two-joint movements, joint torques, muscle synergy

## Abstract

The two-segment model of the human arm is considered; the shoulder and elbow joint torques (JTs) are simulated, providing a slow, steady rotation of the force vector at any end-point of the horizontal working space. The sinusoidal waves describe the JTs, their periods coincide with that of the rotation, and phases are defined by the slopes of the correspondent lines from the joint axes to the end-point. Analysis of the JTs includes an application of the same discrete changes in one joint angle under fixation of the other one and vice versa; the JT pairs are compared for the “shoulder” and “elbow” end-point traces that pass under fixation of the elbow and shoulder angles, respectively. Both shifts between the sinusoids and their amplitudes are unchanged along the “shoulder” traces, whereas these parameters change along the “elbow” ones. Therefore, if we consider a combined action of both JTs acting at the proximal and distal joints, we can assume that for the end-point transitions along the “shoulder,” and “elbow” traces this action possesses *isotropic* and *anisotropic* properties, respectively. The model also determines the patterns of the torques of *coinciding* and *opposing* directions (TCD, TOD), which would evoke a simultaneous loading of the elbow and shoulder muscles with the coinciding or opposing function (flexors, extensors). For a complete force vector turn, the relationship between the TCD and TOD remains fixed in transitions at the “shoulder” end-point traces, whereas it is changing at the “elbow” ones.

## Introduction

Experimental analysis of the central commands that define the parameters of real movements often combine electromyography (EMG) and kinesiology methods. To describe movements of both the entire human body and its separate parts, such as upper and lower limbs, standard approaches of theoretical mechanics are also applied (Hibbeler, 2016). Analysis of multi-joint movements includes the internal models of inter-segmental dynamics (Hollerbach, 1982). Many movement control studies have analyzed relatively fast movements, when velocities of the body segments and their masses are taken into account. To evaluate the central nervous system (CNS) mechanisms for controlling the movements under study, researchers often apply the inverse internal model describing details of biomechanical events (Wolpert and Kawato, 1998; Kawato, 1999; Wolpert and Ghahramani, 2000). Control signals in such a model contain information about the muscle torques defined by inverse dynamics equations. At least partly, the dynamic simulations use the second-order differential equations defining the velocities and accelerations of different limb segments. An alternative method including the theory of position-dependent control (Feldman, 1986, 2011; Bizzi et al., 1992) could be more suitable for the examination of slow movements when the static states of the motor system serve as primary elements of the analysis. An example of this approach is the equilibrium point hypothesis elaborated by Feldman (2011, 2016). The hypothesis assumes that the CNS defines the equilibrium states in the forced interaction of the organism with the environment, while movements constitute transitions between a series of equilibrium states. One of the advantages of the static models is the possibility of accounting for non-linear properties of the neuromuscular system, such as muscle hysteresis (Kostyukov, 1998). Recent studies on various problems of the position-dependent control of the robotic arms can be found elsewhere (Aguilar Ibañez, 2016; Meda-Campana, 2018; Rubio, 2018; Rubio et al., 2018).

Records of slow movements of upper and lower limbs with parallel EMG analysis are frequently used to find the relationships between movements and their central commands. The above approach becomes especially compelling when the same test movements are repeated many times during an experiment in order to apply an off-line averaging procedure. Moreover, this method is suitable for the examination of naturally repeated cyclic movements, such as walking (Bogey and Barnes, 2017) or bicycling (Ting et al., 1999; Wakeling and Horn, 2009). Previously studied examples of voluntarily controlled movements include cyclic planar movements of the arms (Levin et al., 2001) and writing and drawing movements (Dounskaia et al., 2002). Recently, the planar circular movements of the hand with a fixed wrist were studied during the action of elastic tangential loads (Tomiak et al., 2016). Such an experimental model allows one to determine the shoulder and elbow joint torques (JTs) along the movement trajectory, based on the load value and lengths of the limb segments. The above-cited study demonstrates the correspondence between the JTs and the intensities of EMGs recorded from the appropriate muscles. During a complete movement period, each of the JTs includes two components, positive and negative, correlating with activity in the flexor and extensor muscles, respectively. Timings and relative durations of the JTs and EMGs waves are dissimilar for different joints. One of us proposed a simple geometric method that allows us to define the exact positions of the points where the JTs change sign, which simplifies the determination of these points at various curvilinear movement traces in the working space (Kostyukov, 2016). While analyzing two-joint movements, we have also suggested an additional method for marking the sectors of coinciding and opposing synergy along the trajectory of movement (Kostyukov, 2016; Tomiak et al., 2016). The synergy sectors define the sections of the movement trajectory, in which muscles of the same or different function (flexors, extensors) are simultaneously active. A similar procedure for searching the interrelationships between the JTs and EMGs has been applied to the analysis of the isometric muscle contractions when a subject must slowly change the direction of the end-point force in reaction to a visual command signal (Lehedza et al., 2016; Lehedza, 2017).

Following the approaches proposed by Feldman (2011, 2016), the slow (quasi-static) movements are traditionally used to describe the system statics for movement production. In such an approach, the sets of equilibrium states in the system under study usually serve to predict its dynamic behavior. Evaluations of the system statics by temporary changes of the JTs (Lehedza et al., 2016) allow for providing a satisfactory prediction of the EMGs in the muscles generating these forces; however, it seems to be difficult to obtain such data for any point in the working space. In this theoretical study, we have tried to model the essential parameters in the positioning of the limb segments that directly influence the JTs. Two important elements were included in the modeling. First, to take into account all possible directions of the generated forces, we used a steady turning of the force vector within a full cycle of its rotation. Second, to simulate the force generation, we have considered the JTs as functions of two variables representing the current values of the joint angles. Standard methods of analysis allowed us to explore the system behavior for two sets of positioning traces with sequential fixation of variables. This approach led us to find the fundamentally important differences in a combined action of the torques for different types of positioning within the working space. At the same time, we comprehend that the model can be applied only to the analysis of the two-joint muscle contractions in isometry; for considering a real arm movement, the inertial properties of the arm segments, as well as the non-linear effects of neuromuscular dynamics, should be taken into account.

## Experimental background and simulation methods

**Figure 1:**
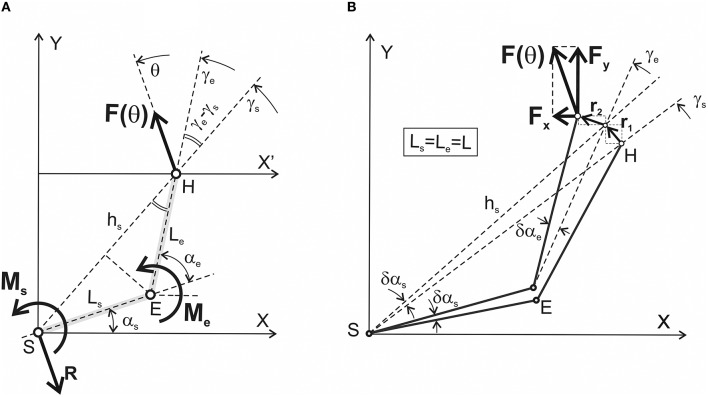
Simplified geometry of the two-joint system with designation of the mechanical parameters defining equilibrium with the surrounding space. **(A)** A right human arm including the shoulder (S) and elbow (E) joints is chosen as an anatomical analog of the system; a similar two-link configuration is used as a basic element in the models of the robotic arm. A simplified forced interaction of the hand (H) with the environment is considered for the case of rigid wrist immobilization. Main characteristics of the system: L_s_, L_e_ are the lengths of the proximal (shoulder) and distal (elbow) segments; **F**(θ) is the vector of the isometric force presenting the result of the interaction of the joint torques M_s_(θ) and M_e_(θ), which are directed perpendicularly to the plane upwardly/downwardly for counter clockwise/clockwise turning actions. The problem consists in finding the joint torques M_s_(θ) and M_e_(θ) for all possible directions of the force vector **F**(θ) (θ∈[0, 2π] rad). Other designations: h_s_ is the distance between the shoulder joint axis (S) and the hand (H), which is considered the end-point; γ_s_ and γ_e_ are the angles between axis X' and the lines passing via the axes of the joints (S, E) and the end-point (H). The force reaction of the body at the shoulder joint is shown by the vector ***R*** = –**F**(θ). **(B)** Graphical presentation of the method of virtual work used to define the functional interdependence between the generated force and joint torques. A detailed description is presented in the text; note that the simulations in this study have been done under a simplifying assumption that lengths of the proximal and distal segments are equal to each other: L_s_ = L_e_ = L.

**Figure 2:**
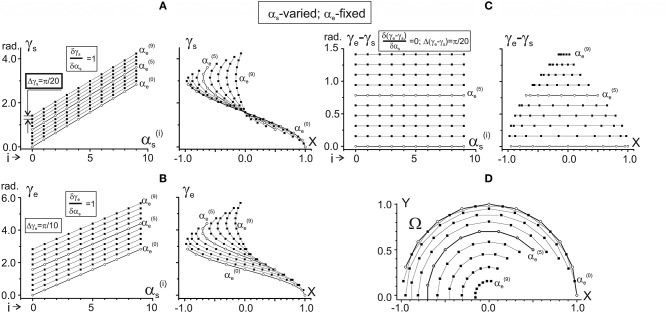
Changes in the characteristic angles γ_s_ and γ_e_ and their difference in dependence on α_s_ provided that α_e_ is fixed. Steps of changes in both arguments are chosen as π/10 rad; separate dependences from α_s_ are drawn for 10 fixed values of α_e_, ranging from $\alpha_{\text{e}}^{\left(0\right)}$ = 0 to $\alpha_{\text{e}}^{\left(9\right)}$ = 9π/10 rad. Successive procedures of numerical analysis are presented in Table 1, left column. The right columns in **(A–C)** present the same data as in the left ones after changing the end-point angles by their projections on the X-axis of the working space Ω (shown in **D**). For better distinguishing, the traces $\alpha_{\text{e}}^{\left(0\right)}$ and $\alpha_{\text{e}}^{\left(5\right)}$ are marked by open circles. For purposes of the data treatment, additional quantitative information about slopes of the characteristics and distances between them is placed in squares at the correspondent panels. Note the same ordinate calibrations in the paired plots of **(A–C)** and the conformity between the points in these plots and their positioning within the working space Ω **(D)**. All dimension characteristics in this and other figures are normalized with respect to the radius of the working space (*R* = 2 L = 1).

Figure 1 schematically describes a process of generation of isometric force by the human hand with an immobilized wrist. The distal segment is interpreted as an “elongated” forearm; the arm and the force vector are located within the horizontal plane passing via the shoulder joint. In experimental studies of the two-joint isometric arm contractions (Lehedza et al., 2016; Lehedza, 2017), the subject's hand grips the top part of a rigid vertical manipulandum, which allows the researcher to register the direction and amplitude of the created force. Lehedza et al. (2016) describe a construction of the manipulandum in detail. The position of the manipulandum can be changed within the working space before a subject; the correspondent hand location coincides with the end-point position of the generated force. In such experimental setups, lengths of the arm segments do not usually differ significantly from each other; the possible difference is not more than 5–7% of the shoulder segment length; therefore, for the sake of simplicity, the segments are assumed to be of the same length (L_s_ = L_e_ = L). The first letters of the “shoulder” (S) and “elbow” (E) terms designate the proximal and distal joints the joint angles (α_s_, α_e_), the lengths of segments (L_s_, L_e_), and the torques (M_s_, M_e_). Therefore, our task consists in searching the torques M_s_ and M_e_, which are necessary to create in the proximal (S) and distal (E) joints to generate the force vector **F**(θ) by the hand in the end-point belonging to the working space Ω (Figure 2). The forces created by the hand could vary in both their amplitude and direction; when the angle argument θ is changed from 0 to 2π radians, the force vector **F**(θ) is turning in the counter clockwise direction. The JTs M_s_ and M_e_ are generated by the cooperative action of the shoulder and elbow muscles. However, we do not consider a possible co-activation of the antagonistic muscles belonging to each of the joints. It is assumed that the force amplitude |**F**| and the length of segments (L) are constant, so the problem consists in finding the JTs as function of the angles θ, α_s_, and α_e_. For a given force vector, the maximal effectiveness of the muscles participating in its creation corresponds to a full inactivity of their antagonists; any contraction of the antagonists would diminish the forced action of the agonists. The co-activation introduces indeterminacy in the system behavior; the co-activation extent can be defined only in a real experiment.

For computer simulations and graphical plotting, we used Origin 8.5 software (OriginLab Corporation, USA). The formulae were computed using the internal language of the software, based on operations with the worksheets; the used worksheets consisted of 1,000 rows and from 6 to 15 columns. To change sets of the fixed parameters in the formulae, we used the replication of basic worksheets.

## Results of modeling

### Determination of the JTs by the virtual work method

To determine the JTs M_s_ and M_e_, which a subject creates by activation of the corresponding muscles acting around the proximal and distal joints, we used the method of virtual work described in detail in textbooks on theoretical mechanics [for example, (Hibbeler, 2016)]. Thus, the problem is to find the sum of works produced by the JTs M_s_ and M_e_ during virtual infinitesimal changes in the joint angles δα_s_ and δα_e_ (Figure 1A). On the other hand, this summed work may be equalized to work produced by the force **F**(θ) along the corresponding path vector **r**, presenting a sum of the two consecutive infinitesimal vectors, **r**_1_ and **r**_2_ (Figure 1B):

(1) $${\text{M}}_{\text{s}}\delta \alpha_{\text{s}}+{\text{M}}_{\text{e}}\delta \alpha_{\text{e}}=\text{F}\cdot \left({\text{r}}_{\text{1}}+{\text{r}}_{\text{2}}\right)=\text{F}\cdot \text{r}={\text{F}}_{\text{x}}{\text{r}}_{\text{x}}+{\text{F}}_{\text{y}}{\text{r}}_{\text{y}}.$$

Figure 1B defines the projections of the force and transition vectors on the coordinate axes:

(2) $$\begin{array}{r} {\text{F}}_{\text{x}}=\text{F cos}\theta ;\;{\text{F}}_{\text{y}}=\text{F sin}\theta ;\;{\text{r}}_{\text{x}}{=\text{r}}_{1\text{x}}+{\text{r}}_{2\text{x}};\;{\text{r}}_{\text{y}}={\text{r}}_{1\text{y}}+{\text{r}}_{2\text{y}}. \end{array}$$

The first transition presented by vector **r**_1_ corresponds to a fixed α_e_; in this case, h_s_ turns on the angle δα_s_. The second transition **r**_2_ coincides with turning the distal segment on the angle δα_e_. Due to small values of δα_s_ and δα_e_, the lengths of the arcs correspond closely to the lengths of vectors **r**_1_ and **r**_2_:

(3) $$\begin{array}{r} {\text{r}}_{1}={\text{h}}_{\text{s}}\delta \alpha_{\text{s}};\;r_{2}=\text{L}\delta \alpha_{\text{e}}. \end{array}$$

Following Figure 1A, it is possible to define the distance h_s_ between the shoulder axis S and the end-point H:

(4) $${\text{h}}_{\text{s}}=\text{L}\left[\text{cos}\left(\gamma_{\text{s}}-\alpha_{\text{s}}\right)+\text{cos}\left(\gamma_{\text{e}}-\gamma_{\text{s}}\right)\right].$$

Due to the importance of the angles γ_s_ and γ_e_ for further considerations, we will call them the characteristic angles (CAs). The following expressions define these parameters:

(5) $$\begin{array}{r} {\text{γ}}_{\text{s}}=\tan^{-1}\left[\frac{sin + sin\left(\alpha_{\text{s}}+\alpha_{\text{e}}\right)}{\text{cos}\alpha_{\text{s}}+\text{cos}\left(\alpha_{\text{s}}+\alpha_{\text{e}}\right)}\right];\;\gamma_{\text{e}}=\alpha_{\text{s}}+\alpha_{\text{e}}. \end{array}$$

The slopes of vectors **r**_1_ and **r**_2_ to the abscissa and ordinate axes are equal to γ_s_+π/2; γ_e_+π/2 and γ_s_; γ_e_, respectively. Therefore, we can find projections of the vector **r** on the coordinate axes:

(6) $$\begin{array}{l} {\text{r}}_{\text{x}}=-{\text{h}}_{\text{s}}\delta \alpha_{\text{s}}\text{ sin}\gamma_{\text{s}}-{\text{L}}_{\text{e}}\delta \alpha_{\text{e}}\text{ sin}\gamma_{\text{e}};{\text{r}}_{\text{y}}={\text{h}}_{\text{s}}\delta \alpha_{\text{s}}\text{ cos}\gamma_{\text{s}}\\ +{\text{L}}_{\text{e}}\delta \alpha_{\text{e}}\text{ cos}\gamma_{\text{e}}. \end{array}$$

After applying appropriate substitutions, Equation (1) is as follows:

(7) $$\begin{array}{l} {\text{M}}_{\text{s}}\delta \alpha_{\text{s}}+{\text{M}}_{\text{e}}\delta \alpha_{\text{e}}=-\text{F cos}\theta \left({\text{h}}_{\text{s}}\delta \alpha_{\text{s}}\text{ sin}\gamma_{\text{s}}+\text{L}\delta \alpha_{\text{e}}\text{ sin}\gamma_{\text{e}}\right)\\ +\text{F sin}\theta \left({\text{h}}_{\text{s}}\delta \alpha_{\text{s}}\text{ cos}\gamma_{\text{s}}+\text{L}\delta \alpha_{\text{e}}\text{ cos}\gamma_{\text{e}}\right). \end{array}$$

Using proper trigonometric conversions, Equation (7) is transformed as follows:

(8) $$\begin{array}{r} {\text{M}}_{\text{s}}\delta \alpha_{\text{s}}{+\text{M}}_{\text{e}}\delta \alpha_{\text{e}}=\text{F}{\text{h}}_{\text{s}}\text{sin}\left(\text{θ}-{\text{γ}}_{\text{s}}\right)\;\delta \alpha_{\text{s}}+\text{F}\mathrm{Lsin}\left(\text{θ}-{\text{γ}}_{\text{e}}\right)\;\delta {\text{α}}_{\text{e}}. \end{array}$$

Finally, we can write apparent expressions for the shoulder and elbow JTs:

(9) $$\begin{array}{r} {\text{M}}_{\text{s}}=\text{F}{\text{h}}_{\text{s}}\text{sin}\left(\theta -\gamma_{\text{s}}\right)\;;{\text{M}}_{\text{e}}=\text{FLsin}\left(\theta -\gamma_{\text{e}}\right). \end{array}$$

Therefore, the combined action of JTs in both joints completely and uniquely determines the amplitude and direction of the end-point force **F**(θ). Within a complete cycle of the force angle change (0 ≤ θ ≤ 2π), two sinusoids describe changes in the shoulder and elbow JTs at a given end-point. The sinusoids have the same period coinciding with the period of the force angle turning; the CAs γ_s_ and γ_e_ define shifts of the sinusoids to the beginning position of the force vector (θ = 0). The elbow JT has an unchanged amplitude FL within the entire working space, while the amplitude of the shoulder JT is changed from 2FL (for a completely extended elbow joint) to zero (in an “idealized” case of a completely flexed elbow joint). In difference from the previous models of the human arm (Feldman, 1986, 2011; Bizzi et al., 1992; Wolpert and Kawato, 1998; Kawato, 1999), the present model describes the patterns of the JTs for the end-point traces that pass under consecutive fixation of the elbow and shoulder angles. Such an approach allows obtaining a simple graphical form of the JTs presentations, what can be highly effective for a preliminary evaluation of the characteristics of the two-joint movements in real experiments.

### Dependence of the CAs on the joint angles

**Figure 3:**
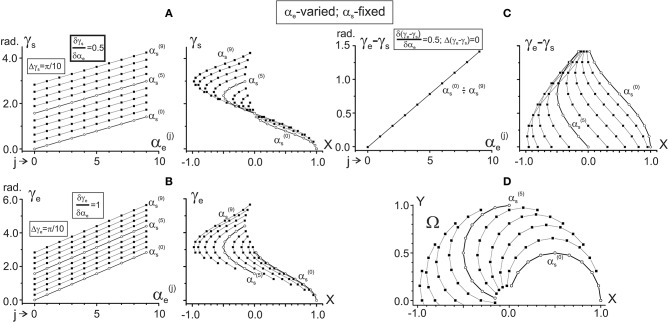
Changes in the characteristic angle*s* γ_s_ and γ_e_ and their difference in dependence on α_e_ provided that α_s_ is fixed. The steps of the joint angles are the same as in Figure 2; successive procedures of numerical analysis are presented in Table 1, right column. The right columns in **(A–C)** present the same data as in the left ones after changing the end-point angles by their projections on the X-axis of the working space Ω (shown in **D**). For ease of distinguishing, the traces $\alpha_{\text{s}}^{\left(0\right)}$ and $\alpha_{\text{s}}^{\left(5\right)}$ are marked by open circles. Quantitative information about slopes of the characteristics and distances between them is placed in squares at the correspondent panels. Note the same ordinate calibrations in the paired plots of **(A–C)** and the conformity between the points in these plots and their positioning within the working space Ω **(D)**.

Standard methods, allowing one to analyze the CAs γ_s_ and γ_e_ as functions of two variables α_s_ and α_e_ (see Equation 5), include determination of their dependencies on each of the arguments when another one is fixed. Therefore, two pairs of the functions should be considered: (1) γ_s_(α_s_|α_e_ = const); γ_e_(α_s_|α_e_ = const), and (2) γ_s_(α_e_|α_s_ = const); γ_e_(α_e_|α_s_ = const). Successive procedures of the numerical analysis, based on the equations of the previous section, are presented in Table 1; the results of the simulations are shown in Figures 2, **3**. Figure 2 describes the CAs γ_s_ and γ_e_, as well as their difference (γ_e_ – γ_s_), which are defined depending on α_s_ for fixed values of α_e_. Figure 3 presents similar data based on an opposite relationship between the varying and fixed arguments.

**Table 1:** Sequences of the procedures used to determine the *characteristic angles* γ_e_ and γ_s_ in various end-point positions within the working space.

	**“Shoulder” end-point traces**	**“Elbow” end-point traces**
	${{\text{fixed}:\;\alpha }_{\text{e}}}^{\left(\text{K}\right)}=\text{K}\frac{\pi }{10};\text{ K}=0\;\ldots \;9$; ${{\text{varying}:\;\alpha }_{\text{s}}}^{\left(\text{i}\right)}=\text{i}\frac{\pi }{10},\text{ i}=0\;\ldots \;9;$	${{\text{fixed}:\;\alpha }_{\text{s}}}^{\left(\text{L}\right)}=\text{L}\frac{\pi }{10};\text{ L}=0\;\ldots \;9;$ ${{\text{varying}:\;\alpha }_{\text{e}}}^{\left(\text{j}\right)}=\text{j}\frac{\pi }{10},\text{ j}=0\;\ldots \;9;$
1	$\text{x}\left(\text{i}|\text{K}\right)=0.5\left[\text{cos}\left({\alpha_{\text{s}}}^{\left(\text{i}\right)}\right)+\text{cos}\left({\alpha_{\text{s}}}^{\left(\text{i}\right)}+{\alpha_{\text{e}}}^{\left(\text{K}\right)}\right)\right];$ $\text{y}\left(\text{i}|\text{K}\right)=0.5\left[\sin\left({\alpha_{\text{s}}}^{\left(\text{i}\right)}\right)+\sin\left({\alpha_{\text{s}}}^{\left(\text{i}\right)}+{\alpha_{\text{e}}}^{\left(\text{K}\right)}\right)\right];$	$\text{x}\left(\text{j}|\text{L}\right)=0.5\left[\cos\left({\alpha_{\text{s}}}^{\left(\text{L}\right)}\right)+\cos\left({\alpha_{\text{s}}}^{\left(\text{L}\right)}+{\alpha_{\text{e}}}^{\left(\text{j}\right)}\right)\right];$ $\text{y}\left(\text{j}|\text{L}\right)=0.5\left[\sin\left({\alpha_{\text{s}}}^{\left(\text{L}\right)}\right)+\sin\left({\alpha_{\text{s}}}^{\left(\text{L}\right)}+{\alpha_{\text{e}}}^{\left(\text{j}\right)}\right)\right];$
2	${\text{h}}_{\text{s}}\left(\text{i}|\text{K}\right)=\sqrt{{\text{x}}^{2}\left(\text{i}|\text{K}\right)+{\text{y}}^{2}\left(\text{i}|\text{K}\right)}$;	${\text{h}}_{\text{s}}\left(\text{j}|\text{L}\right)=\sqrt{{\text{x}}^{2}\left(\text{j}|\text{L}\right)+{\text{y}}^{2}\left(\text{j}|\text{L}\right)}$;
3	$\gamma_{\text{e}}\left(\text{i}|\text{K}\right)-\gamma_{\text{s}}\left(\text{i}|\text{K}\right)=\;\cos^{-1}{\text{h}}_{\text{s}}\left(\text{i}|\text{K}\right)$;	$\gamma_{\text{e}}(\text{j}|\text{L})-\gamma_{\text{S}}(\text{j}|\text{L})={\text{cos}}^{-1}\left[{\text{h}}_{\text{S}}(\text{j}|\text{L})\right];$;
4	$\gamma_{\text{s}}\left(\text{i}|\text{K}\right)=\cos^{-1}\left[\frac{\text{x}\left(\text{i}|\text{K}\right)}{{\text{h}}_{\text{s}}\left(\text{i}|\text{K}\right)}\right];$	$\gamma_{\text{s}}\left(\text{j}|\text{L}\right)=\cos^{-1}\left[\frac{\text{x}\left(\text{j}|\text{L}\right)}{{\text{h}}_{\text{s}}\left(\text{j}|\text{L}\right)}\right];\;$
5	γ_e_(*i*|*K*) = γ_s_(*i*|*K*)+[γ_e_(*i*|*K*)−γ_s_(*i*|*K*)].	γ_e_(*j*|*L*) = γ_s_(*j*|*L*)+[γ_e_(*j*|*L*)−γ_s_(*j*|*L*)].

*The positions are changed along the lines of the fixed elbow and shoulder joint angles (left and right columns, respectively). Pairs of the indexes noted by small (i, j) and capital (K, L) letters belong to the varied and fixed parameters, respectively. Figures 2, 3 describe the results of the simulation*.

One can see that both γ_s_ and γ_e_ depend linearly on each of the arguments, α_s_ and α_e_, although it is possible to point out some essential differences. When comparing the dependencies of the CAs on α_s_ (Figures 2A,B), the slopes of the lines are equal (δγ_s_/δα_s_ = δγ_e_/δα_s_ = 1), and there is a two-fold difference in the distance between adjacent lines (Δγ_s_ = π/20, Δγ_e_ = π/10). On the other hand, the dependencies of the CAs on α_e_ (Figures 3A,B) demonstrate a coincidence of the distances between the lines (Δγ_s_ = Δγ_e_ = π/10), while their slopes show a two-fold difference (δγ_s_/δα_e_ = 0.5; δγ_e_/δα_e_ = 1). Such properties of the CAs lead to essential distinctions in the corresponding behavior of their subtraction. The difference between CAs (γ_e_ – γ_s_) defines a relative shift between the JT sinusoids at various end-point positions within the working space. As shown below (section Patterns of Activation of the Proximal and Distal Muscles), such a shift is the primary parameter influencing the torque patterns of the muscles belonging to different joints. In other words, in the two-joint movements, the difference between CAs directly affects the interaction of activity in the muscles of different joints.

The dependency of (γ_e_ – γ_s_) on α_s_ remains constant for any fixed value of α_e_, and it linearly rises with the α_e_ increase (Figure 2C). It should be noticed that a linear increase in the CA difference (γ_e_ – γ_s_) with a rise in α_e_ under fixed values of α_s_ is associated with a complete coincidence of the separate lines belonging to different α_s_ (Figure 3C). A definite interest may present the traces of the CA differences, which are plotted against the frontal coordinate of the end-point position (compare right panels in Figures 2C, **3C**). For fixed values α_e_ (Figure 2C), these traces present horizontal lines, shifting in an upward direction with a rise of α_e_. In contrast, when α_s_ is fixed, the correspondent traces have a complex curvilinear appearance, which changes with the increase in α_s_ (Figure 3C). Therefore, one can see that the torque patterns are not changed in the first case and demonstrate a complex modification in the second one.

The joint angles α_s_, α_e_ are defined unambiguously for any end-point within the working space. Thus, it is possible to change arguments in plots γ_s_(α_s_), γ_e_(α_s_) and γ_s_(α_e_), and γ_e_(α_e_), which are shown in the left panels in Figures 2A,B, 3A,B, replacing the joint angles by projections of the correspondent points on the X-axis. The right panels in Figures 2A,B, 3A,B demonstrate the results of such a change in the variables. The sets of points in the plots γ_s_(X) presented in Figures 2A, 3A coincide with each other; the only difference relates to the lines connecting the points at these plots. The discrepancy between the lines is due to a difference in the varying and fixed arguments in both sets (compare Figures 2D, 3D). Two sets of the plots γ_e_(X) presented in Figures 2B, 3B show similar behavior. The sets of the points γ_s_(X) and γ_e_(X) demonstrate both similarities and differences. The similarities consist in the likeness of the point distributions, both of which take up more areas at the left part of the working space. The differences lie in the observation that, at the right part of the working space, the γ_e_(X) points are distributed over a relatively broader area compared with the γ_s_(X) points. Such a distribution is mainly well seen near the position of the shoulder joint axis (X = 0). We also note that the γ_e_(X) points cover a more significant range of the angles, compared with the γ_s_(X) ones (about six radians vs. four).

### Dependence of the JTs on the force direction

By using the above CA plots, it is possible to analyze the JTs at various angles of the end-point force. For simplicity, we do not take into account potential problems associated with the existence of two-joint muscles or with the co-activation of the muscle-antagonists. The controlled changes in the direction of the isometric force vector (change of angle θ in Figure 1A) are realized in our experimental conditions as follows [for details see (Lehedza et al., 2016; Lehedza, 2017)]. A subject creates with his right hand isometric pressure on an unmovable handle, allowing one to measure both the amplitude and direction of the generated force. When performing a task of visual tracing of the force vector, a subject slowly changes the force vector direction under the command signal specified by a point slowly moving along a circular trace on the monitor screen. The center of the circle corresponds to the human's hand position; its radius defines the force amplitude.

**Figure 4:**
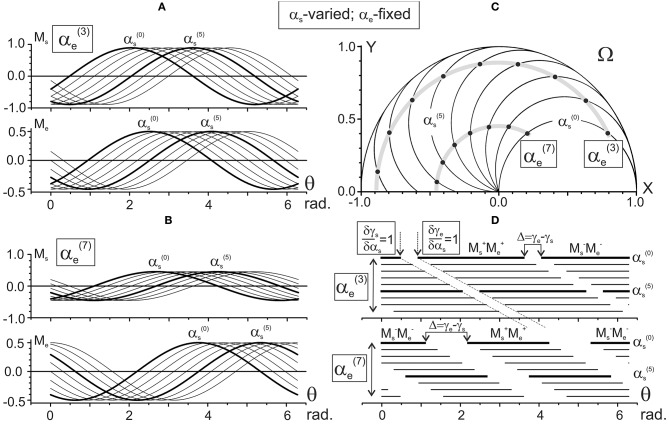
Results of numerical simulation of the joint torques M_s_ and M_e_ **(A,B)** for different positions of the subject's hand in the working space Ω **(C)**. Two sets of the torque records with fixation of the elbow joint angle in positions 3π/10 [$\alpha_{\text{e}}^{\left(3\right)}$] and 7π/10 [$\alpha_{\text{e}}^{\left(7\right)}$] rad are chosen. The torques M_s_ and M_e_ are defined by Equation (9) using the characteristic angles presented in Figure 2A. Thicker lines highlight the torque traces for shoulder positions $\alpha_{\text{s}}^{\left(0\right)}$ and $\alpha_{\text{s}}^{\left(5\right)}$. Horizontal lines in **(D)** mark phases of the sign coincidence of the shoulder and elbow torques, both positive (M${}_{\text{s}}^{+}$M${}_{\text{e}}^{+}$) and negative (M${}_{\text{s}}^{-}$ M${}_{\text{e}}^{-}$), in different traces. Throughout the study, the torques are defined for the action of unit forces at the hand positions; therefore, their calibrations are given in arbitrary units.

**Figure 5:**
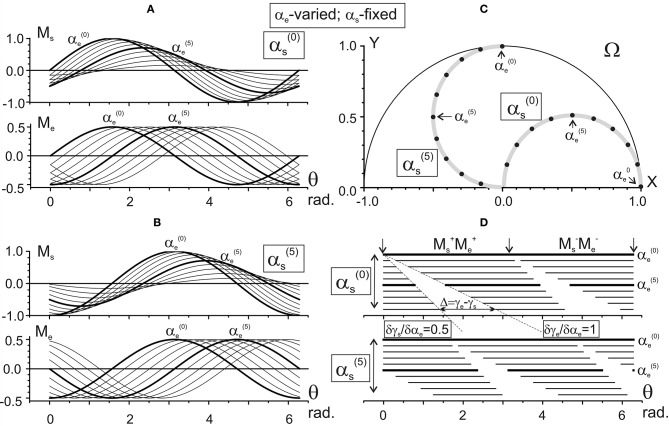
Results of the numerical simulation of the joint torques M_s_, M_e_ **(A,B)** for different positions of the subject's hand in the working space Ω **(C)**. Scheme of the data presentation coincides with that in Figure 4; two sets of the torques with fixation of the shoulder joint angles $\alpha_{\text{s}}^{\left(0\right)}$ and $\alpha_{\text{s}}^{\left(5\right)}$ are considered The torques M_s_ and M_e_ are defined by Equation (9) using the characteristic angles presented in Figure 2B. Thicker lines highlight the torque traces for the elbow joint angles $\alpha_{\text{e}}^{\left(0\right)}$ and $\alpha_{\text{e}}^{\left(5\right)}$. Horizontal lines in **(D)** mark phases of the sign coincidence of the shoulder and elbow torques. Other designations are similar to those in Figure 4.

Figures 4, 5 present the results of computing the JTs M_s_ (θ) and M_e_ (θ) for different positions of the subject's hand. Figure 4 demonstrates the changes in the JTs' dependencies on α_s_ for two fixed elbow positions, $\alpha_{\text{e}}^{\left(3\right)}$ (Figure 4A) and $\alpha_{\text{e}}^{\left(7\right)}$ (Figure 4B). The M_s_ and M_e_ families of curves in Figures 4A,B contain the sinusoids that are consecutively shifting to the right with a rise in their order, and the shifts are equal for both joints. Such a picture corresponds to the equality of the gradients of both CAs with respect to the α_s_ (δγ_s_/δα_s_ = δγ_e_/δα_s_ = 1) (Figure 2A). At the same time, a change in the fixed parameter [i.e., $\alpha_{\text{e}}^{\left(3\right)}$→$\alpha_{\text{e}}^{\left(7\right)}$ in Figures 4A,B] evokes different shifts of both sets of curves while keeping a distance between the curves in each of the sets. The M_s_ sets of curves shift twice as slowly as the M_e_ ones (Δγ_s_ = π/20; Δγ_e_ = π/10 in Figures 2A,B). While comparing two M_s_ sets relating to different values of the shoulder angle [$\alpha_{\text{e}}^{\left(3\right)}$ and $\alpha_{\text{e}}^{\left(7\right)}$ in Figures 4A,B], one can notice a drop in the torque amplitudes, which corresponds to a shortening of the torque arm h_s_ (see Equation 9). In contrast, the M_e_ amplitudes remain unchanged due to the steadiness of the similar parameter coinciding with the segment's length L.

Figure 5 demonstrates the elbow angle-dependent changes of the JTs. In contrast to the above-described changes, the amplitudes of the M_s_ curves change in this case even within the same set (Figures 5A,B). The interval between curves in the M_s_ sets is half that of the M_e_ ones, which is due to a correspondent inequality in the slopes of CAs (δγ_s_/δα_e_ = 0.5; δγ_e_/δα_e_ = 1, see Figures 3A,B).

### Patterns of activation of the proximal and distal muscles

**Figure 6:**
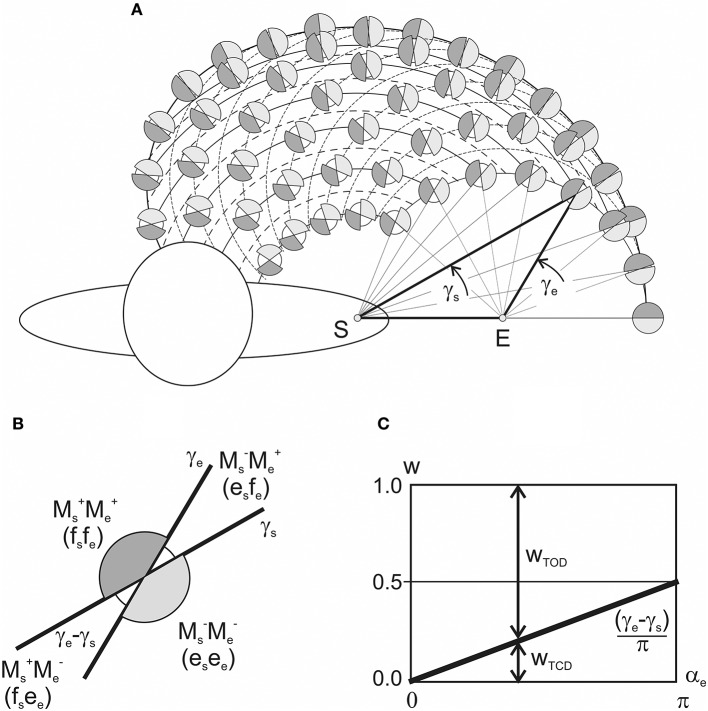
Schematic presentation of the patterns of the torques of *coinciding* and *opposing* directions (TCD, TOD) in dependence on the positioning of the end-points within the working space. **(A)** The torque combinations are depicted by the shadowed (TCD) and white (TOD) sectors at the circles interposed in the nodes of intersection of the “shoulder” and “elbow” end-point traces with fixed values of α_e_ and α_s_, respectively. **(B)** Combinations the joint torques and related patterns of loading of the flexor (f) and extensor (e) muscles belonging to different joints. **(C)** Changes in the TCD and TOD weights in dependency on the elbow joint angle (plot in accordance with Equation 10).

Schematic presentation of various combinations of the activity of flexor and extensor muscles belonging to different joints, as is shown in Figures 3D, 4D, allows us to present in graphical form the changes in the torque patterns (TCD, TOD) for separate transition movements in the joints. As has been shown for two-joint circular movements under a tangential load, central commands to the muscles depend predominantly on positions of the force singular points (FSP), where the JTs change their directions (Kostyukov, 2016; Tomiak et al., 2016). In the above-cited studies, the torque patterns in two-joint movements are considered through the functions of the simultaneously contracted muscles that belong to different joints. The TCD corresponds to contractions of the muscles of the same function (flexors–flexors; extensors–extensors), while the TOD belongs to combinations of the muscles of the opposite modalities (flexors–extensors; extensors–flexors). The proposed approach allows us to analyze the torque patterns for isometric contractions using the CAs (Figure 6). For changes in the force vector angle from 0 to 2π rad, the lines, which are used to designate the CAs, γ_s_ and γ_e_, define two pairs of the torque sectors: TCD (M${}_{\text{e}}^{+}$M${}_{\text{s}}^{+}$, M${}_{\text{e}}^{-}$M${}_{\text{s}}^{-}$) and TOD (M${}_{\text{e}}^{+}$M${}_{\text{s}}^{-}$, M${}_{\text{e}}^{-}$M${}_{\text{s}}^{+}$) (Figures 6A,B). The weights of the torque sectors (see Figures 6C) are defined as follows:

(10) $${\text{w}}_{\text{TOD}}=\frac{\left(\gamma_{\text{e}}-\gamma_{\text{s}}\right)}{\pi };{\text{w}}_{\text{TCD}}=1-\frac{\left(\gamma_{\text{e}}-\gamma_{\text{s}}\right)}{\pi }.$$

The maximal weight of the TCD, equal to 1, relates to a fully extended elbow joint (α_e_ = 0) for any α_s_. A rise in the α_e_ decreases the TCD weight linearly, converging to a limit value of 0.5 in a hypothetical case of a complete joint flexion (α_e_ = 180°), whereas the weight of the TOD rises from 0 to 0.5 during an increase in the α_e_ from 0 to 180°. Therefore, one can conclude that in movements around the shoulder joint, the torque patterns remain invariable; at the same time, they are noticeably dependent on the elbow joint angles. During a rise in the α_e_, the weights of TCD and TOD change linearly in the opposite direction, whereas the relationship between them remains unvaried for any fixed α_e_.

## Discussion

The JTs that accompany generation of forces by the human right hand are simulated in our study in a framework of a two-joint model of the right arm placed horizontally. The simulation is based on a method of virtual work [for example, see (Hibbeler, 2016)] that had allowed us to define the JTs at each of two joints for any direction of the end-point force and position in the working space. When the frontal slopes of the force vectors (angle θ) change in the range 0–2π, the JTs M_s_ and M_e_ are presented in dependency on the angle by the sinusoidal functions of different amplitudes and phase lags. The CAs γ_s_ and γ_e_ define the phase lags of the sinusoids; the elbow JTs are not changed, being equal to the product of the force amplitude and segment length FL; the shoulder JTs, equal to Fh_s_, vary with the distance from the axis of the shoulder joint to the end-point, h_s_. For a complete cycle of the force vector turning, the relative times of the flexor and extensor contractions in each of the joints are equal. From the basic geometric definitions, it follows that γ_e_ ≥ γ_s_ for the entire working space (see Figure 1). Therefore, during continuous turning of the end-point force vector in the counter clockwise direction, the shoulder flexors should always be activated earlier than the elbow flexors, and this has been demonstrated experimentally (Lehedza et al., 2016; Lehedza, 2017).

**Figure 7:**
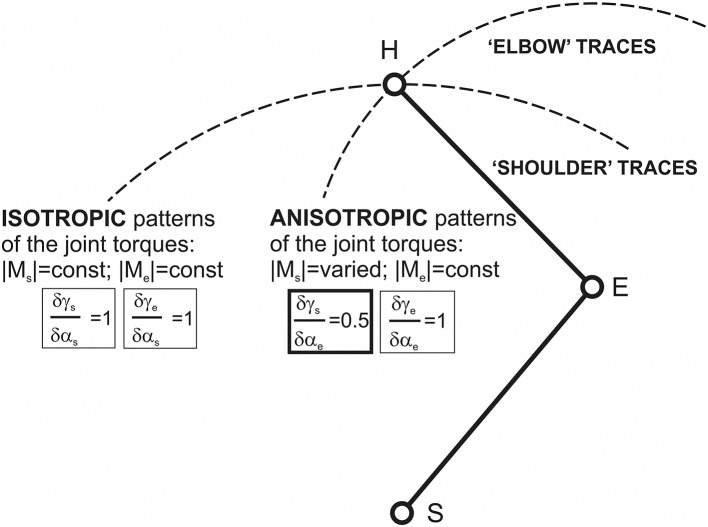
Schematic description of possible differences given changes in the central commands to muscles during the end-point transitions along “shoulder” and “elbow” traces. These differences can be strongly connected with related patterns of the JT changes: isotropic in the first case and anisotropic in the second. Isotropy and anisotropy in changes to the JTs along the “shoulder” and “elbow” traces could be directly related to the equality (inequality) of the relative changes to the CAs γ_s_ and γ_e_ with respect to the correspondent joint angles.

When considering the isometric muscle contractions for different end-point positions in the curvilinear coordinate system {α_s_; α_e_}, it is entirely reasonable to evaluate changes of the shoulder and elbow torque waves for isolated changes in the joint angles, i.e., during the end-point transitions along the “shoulder” and “elbow” traces (Figure 6). The gradients of the phase shifts for the both M_s_ and M_e_ waves coincide with each other along the “shoulder” traces: δγ_s_/δα_s_ = δγ_e_/δα_s_ = 1, while along the “elbow” traces, the M_s_ phases shift half as fast as the M_e_: δγ_s_/δα_e_ = 0.5; δγ_e_/δα_e_ = 1 (Figure 7). Taking into account experimental findings of the correspondence between the timings of the EMGs and related parts of the JTs waves (Lehedza et al., 2016; Lehedza, 2017), the above results may be applied to predict the shifts of the central commands for the respective muscle contractions (Figure 7). In the end-point transitions along the “shoulder” traces (α_s_-varying; α_e_-fixed), shifts between M_e_ and M_s_ waves remain unchanged; therefore, the torque waves are changing in an *isotropic* manner. In contrast, for the end-point transitions connected with the shoulder joint and fixed elbow one, the torques waves demonstrate the *anisotropic* manner of changing.

The central commands to the muscles in two-joint movements depend predominantly on the relative positions of FSPs, where the JTs change their directions (Lehedza et al., 2016; Tomiak et al., 2016; Lehedza, 2017). The FSPs may be used to identify different zones of the torques of *coinciding* and *opposing* directions (TCD, TOD), which would evoke a simultaneous loading of the elbow and shoulder muscles with the coinciding or opposing function (flexors, extensors). The distribution of the CA difference (γ_e_ – γ_s_) in the working space defines the TCD and TOD sectors (Figure 6). A maximal weight of the TCD (equal to 1) corresponds to a fully extended elbow joint (α_e_ = 0) for any α_s_-value. The weight is linearly decreased with a rise of α_e_, converging to a limit value 0.5 at the hypothetical case of the complete flexed elbow, α_e_ = 180°. Contrastingly, the TOD weight rises from 0 to 0.5 during the α_e_ increase of from 0 to 180°. Therefore, the torque patterns are not changed for the isolated movements around the shoulder joint, being, at the same time, noticeably dependent on the elbow joint angles. During a rise of the α_e_, weights of the TCD and TOD change linearly in opposite directions; however, for fixed α_e_, the relationship between the torque patterns remains unvaried for all α_s_-values (Figure 6B). A predominance of the TCD effects for the entire working space can exert an essential influence on the central commands to the muscles. If we assume an equal probability for all possible directions of the end-point forces in a variety of movement programs, one can encounter more frequently the associations of descending activities to the muscles of the same function in different joints (i.e., flexors–flexor or extensors–extensors). The predominance of the TCD effects becomes more and more pronounced with the increase in the end-point distances from the proximal joint; and their maximal weight is achieved at the circular boundary of the working space (Equation 10; Figure 6C). Such a pattern of the torque effects can provide some simplification of both descending motor programs and their realization at the spinal level. At the same time, the above inferences might be related only to a restricted class of movement tasks associated with a generation of the isometric forces **F**(θ) in all possible directions (0 ≤ θ < 2π) and locations of the end-points within the working space.

The present study includes the analysis of the steady states in two-joint movements, whereas real fast movements are inevitably much more complicated and diversified. Directional preferences in the arm movements were previously revealed for horizontal arm movements and interpreted by a simplified joint control program that involves predominantly passive motion at either the shoulder or elbow (Dounskaia and Goble, 2011; Dounskaia et al., 2011; Dounskaia and Wang, 2014). In studies of skilled throwing in baseball, Hirashima et al. (2007) supported the idea that the CNS could control complex movements by using a hierarchical strategy such as described by the *leading joint hypothesis* proposed by Dounskaia (2005). The theory suggests that planning of complex movement becomes simpler by choosing one “leading” joint, which provides the dynamic foundation for the entire movement. The kinematics of the leading joint is controlled actively with agonist-antagonist muscle activity similar to that used for the control of single-joint movements. The adjacent “subordinate” joint is strongly influenced by passive dynamics, with activity in the “subordinate” muscles used to adjust the joint kinematics to meet the requirements of the task. In two-joint arm movements, the shoulder joint is usually considered the “leading” one due to a large volume of the musculature and higher inertia of the upper arm. However, fast movements, in which the elbow plays the leading role while the shoulder is subordinated, have been described as well (Debicki et al., 2011).

Subjects can produce arm movements with different speeds and trajectories. In general, however, it is unclear how the CNS plans and coordinates shoulder and elbow motions. The so-called “interaction torques” participate in fast movements, which arise at one joint due to the rotation of adjacent joints (Hollerbach and Flash, 1982). For example, rotation of the proximal shoulder joint influences the motion of the distal elbow and wrist joints through interaction of the torques in the proximal-to-distal direction; similarly, rotation of the distal joints can influence proximal joint motion (Latash et al., 1995; Gribble and Ostry, 1998; Dounskaia et al., 2002; Debicki et al., 2011). On the other hand, in statics (during isometric contractions or slow movements), it seems possible to exclude the above intersegmental interactions. In difference from existing models of the two-joint movements, we concentrated main attention on the positioning of the end-point force vector within the working space. Such an approach allows finding the patterns of the JTs that provide various slopes of the generated efforts in any point of the space. As follows from the present consideration, it is possible to evaluate the interdependence between the end-point force and the JTs at the both joints.

We would like to stress that any consideration of the equilibrium states in two-joint arm movements must also take into account the numerous non-linear properties of a transformation of the efferent signals to muscle contraction. At least three essential elements of uncertainty are present in the static states of the arm under given conditions of loading. First, the prehistory of activation and movement strongly affects the steady states in the system; these processes are directly related to muscle hysteresis (Kostyukov, 1998). Second, both agonist and antagonist muscles provide the resultant torque in each joint; co-activation of the antagonists can constitute a substantial source for the uncertainty in the equilibrium states of the joints (Gorkovenko et al., 2012). Third, the redistribution activity among different parts of individual muscles and between different muscles can be highly expressed, which inevitably leads to ambiguity of motor control.

## Conclusions

The two-segment model of the human arm simulates the shoulder and elbow JTs, providing a slow, steady rotation of the force vector in any end-point of the horizontal working space. The model can be only applied to the analysis of the two-joint muscle contractions in isometry; for considering a real arm movement, the inertial properties of the arm segments, as well as the non-linear effects of neuromuscular dynamics, should be taken into account.

For the force vector slowly rotating at a constant speed, two sinusoidal waves of the same period, equal to that of rotation, describe the elbow and shoulder JTs; the phases of the sinusoids coincide with the slopes of the correspondent lines from the joint axes to the end-point.

For the analysis of the JTs, we propose considering the “shoulder” and “elbow” end-point traces, in which the correspondent joint angle changes under fixation of the other one. Both shifts between the shoulder and elbow JTs and their amplitudes remain unchanged along the “shoulder” tracks, whereas these parameters change essentially at the “elbow” ones. Therefore, the combined action of both JTs possesses *isotropic* and *anisotropic* properties at the “shoulder,” and “elbow” traces, respectively.

The proposed model determines the patterns of the TCD, TOD, which would evoke a simultaneous loading of the elbow and shoulder muscles with the *coinciding* or *opposing* function (flexors, extensors). The relationship between the TCD and TOD remains fixed in transitions at the “shoulder” end-point traces, whereas it is changing at the “elbow” ones.

## Author contributions

AK: the idea for the study and computer simulations, manuscript writing; TT: discussion of the results, organization of the financial support for the project.

### Conflict of interest statement

The authors declare that the research was conducted in the absence of any commercial or financial relationships that could be construed as a potential conflict of interest.

## Abbreviations

EMG: electromyography
CNS: central nervous system
JT: joint torque
TCD: torques of coinciding directions
TOD: torques of opposing directions
CA: characteristic angle
FSP: force singular point.
